# “Cheaper and better”: Societal cost savings and budget impact of changing from systemic to intralesional pentavalent antimonials as the first-line treatment for cutaneous leishmaniasis in Bolivia

**DOI:** 10.1371/journal.pntd.0007788

**Published:** 2019-11-06

**Authors:** Daniel Eid Rodríguez, Miguel San Sebastian, Anni-Maria Pulkki-Brännström

**Affiliations:** 1 Department of Biomedical Sciences Research, Faculty of Medicine, San Simon University, Cochabamba, Bolivia; 2 Department of Epidemiology and Global Health, Faculty of Medicine, Umeå University, Umeå, Sweden; Saudi Ministry of Health, SAUDI ARABIA

## Abstract

**Introduction:**

Cutaneous leishmaniasis (CL), endemic in Bolivia, mostly affects poor people in rainforest areas. The current first-line treatment consists of systemic pentavalent antimonials (SPA) for 20 days and is paid for by the Ministry of Health (MoH). Long periods of drug shortages and a lack of safe conditions to deliver treatment are challenges to implementation. Intralesional pentavalent antimonials (ILPA) are an alternative to SPA. This study aims to compare the cost of ILPA and SPA, and to estimate the health and economic impacts of changing the first-line treatment for CL in a Bolivian endemic area.

**Methods:**

The cost-per-patient treated was estimated for SPA and ILPA from the perspectives of the MoH and society. The quantity and unit costs of medications, staff time, transportation and loss of production were obtained through a health facility survey (N = 12), official documents and key informants. A one-way sensitivity analysis was conducted on key parameters to evaluate the robustness of the results. The annual number of patients treated and the budget impact of switching to ILPA as the first-line treatment were estimated under different scenarios of increasing treatment utilization. Costs were reported in 2017 international dollars (1 INT$ = 3.10 BOB).

**Results:**

Treating CL using ILPA was associated with a cost-saving of $248 per-patient-treated from the MoH perspective, and $688 per-patient-treated from the societal perspective. Switching first-line treatment to ILPA while maintaining the current budget would allow two-and-a-half times the current number of patients to be treated. ILPA remained cost-saving compared to SPA in the sensitivity analysis.

**Conclusions:**

The results of this study support a shift to ILPA as the first-line treatment for CL in Bolivia and possibly in other South American countries.

## Author summary

Cutaneous leishmaniasis is a neglected tropical disease which causes chronic skin ulcers and disproportionally affects the poorest of the poor. The current first-line treatment, based on systemic pentavalent antimonials, is quite toxic for patients but also expensive for governments and patients. Given limited budgets, national decision-makers need to know not only the efficacy of alternative treatments, but also how to maximise the population health impact of available resources. To strengthen the evidence base for the scale-up of effective NTD interventions in the era of universal health coverage, we conducted cost-effectiveness analyses and estimations of population-level impact of the implementation of intralesional pentavalent antimonials as first-line treatment for cutaneous leishmaniasis using realistic scenarios. Our results demonstrate that changing first-line treatment would have considerable economic advantages for patients due to the shorter treatment duration (savings in terms of food, accommodation, transport, productivity loss); and for the national control program due to the reduced use of drugs and staff time. In addition, this change would offer better value for money given that two-and-a-half times the current number of patients could be treated with the current expenditure level if intralesional pentavalent antimonials were offered to eligible patients.

## Introduction

Leishmaniasis is a group of parasitic infectious diseases transmitted by the bites of infected sandflies. One of the most common syndromes produced by the *Leishmania* parasite in Latin America, is cutaneous leishmaniasis (CL), characterised by chronic skin ulcers on the site bitten by sandflies. It has been estimated that CL affects more than 300,000 people per year, and 39 million are at risk worldwide [1, 2]. In Bolivia, the country with the fifth most CL cases in the region, 2300 new cases are reported every year [3].

Ulcers can heal spontaneously, studies describe healing times ranging from nine months to over a year [4, 5]. Without treatment, ulcers can increase in size, resulting in severe skin scars and consequently affecting the psychological and social well-being of the affected individual [6]. Social stigmatisation may also occur, mostly in cases where women are affected, and where skin scars are located on visible body areas [7–10].

Similar to other neglected tropical diseases, leishmaniasis mainly affects socially vulnerable populations [11, 12]. Patients with leishmaniasis diseases are commonly characterised by living in poor housing conditions with a lack of sanitation [13, 14]. Their per capita income is usually low, which makes it difficult for them to pay additional costs related to treatment seeking, such as transportation to health centres, food and lodging [4, 15–17]. The care seeking process can be catastrophic for weak household economies and become more complicated due to income loss as a result of the inability to perform physical work [18, 19].

Strategies of vector and reservoir control are not feasible in the majority of the Latin American countries because there are many wild animal species and vectors involved in the transmission chain. Therefore, case detection and treatment is the main control strategy for CL. First line treatment consists of systemic pentavalent antimonials (SPA) administered via intravenous or intramuscular for 20 consecutive days. Intralesional pentavalent antimonials (ILPA) have been recommended by the Pan American Health Organization (PAHO) Experts Committee on the Control of Leishmaniases [20] and World Health Organization (WHO) [21] as an alternative treatment for CL. ILPA is non-inferior compared to SPA in terms of efficacy (77% and 75% respectively) [22, 23]. ILPA has other important advantages compared to SPA, such as its short length (5 days), reduced adverse effects, less contraindications, and the absence of systemic toxicity. As a consequence, with ILPA there is no need to use additional diagnostic aids to detect life-threatening conditions related to SPA toxicity [24–27]. Furthermore, followup studies did not find evidence of mucosal complications using ILPA [28, 29].

The lack of equipment and poor access to health services is a common problem in remote rural areas of the Latin American rainforest [4, 5, 16, 30, 31]. In Bolivia, patients must often wait to receive treatment during long periods of drug shortage as the limited budget of the MoH is not enough to buy the amount of drugs needed. In this regard, ILPA could be a good option in those places where the provision of the SPA is costly and cannot be performed in adequate conditions. The potential cost savings from the shorter length of treatment changing to ILPA as first line treatment may be attractive for health systems that seek to maximise health benefits within a given budget.

However, despite the evidence of its potential usefulness, there is currently no economic study evaluating the use of ILPA for CL treatment which could support the adoption and implementation of these therapies in health systems. Economic studies that transfer the results of clinical trials to realistic scenarios that support the economic decisions of stakeholders are required. Such an analysis should also consider the overall impact on the MoH budget, which depends not only on cost per patient treated but also on whether changes in the affordability of treatment for households also change care-seeking patterns.

The aims of this study are to estimate, in a Bolivian rainforest area: 1) the cost per patient treated with ILPA and SPA respectively; 2) the budget impact of changing to ILPA as first-line treatment; and 3) the additional number of potentially treated patients if ILPA is offered as first-line treatment.

## Methods

### Study settings and population

The study setting is the rural area of the Bolivian rainforest where treatment for CL is provided by primary health centres as part of the public health care system.

The cost of ILPA was compared with the cost of SPA for the target population, which is all patients diagnosed with skin ulcers due to CL who are eligible for ILPA according to the PAHO-WHO guidelines [20], which are: 1) lesion size up to 900 mm2; 2) no more than one lesion; 3) lesions not located on face, joints or genitals; and 4) an absence of immunosuppression. The target population for the budget impact analysis and population-level scenarios of the number of cases treated by changing ILPA to the first-line treatment consisted of all CL patients, of whom a proportion would be eligible for ILPA and the remainder would receive SPA; regardless the sex or age of the patient, or the duration of the ulcers.

The Villa Tunari (VT) municipality was chosen as the study area for the cost analysis. The Isiboro Secure National Park, one of the largest ecological reserves in the country, and a natural habitat of the vectors and reservoirs of *Leishmania* parasites, is in VT. This municipality has one hospital and thirty-two health centres, of which only the hospital can provide SPA treatment in safe conditions with adequate toxicity control evaluations that include a complete blood count, the measurement of urea, creatinine, glucose, electrolytes and liver and pancreatic enzymes, in addition to electrocardiograms [32]. This hospital is located more than 200 km away from the national park. Most communities except those located near the main roads have poor public transport, and many are physically inaccessible during the rainy season. According to the latest population census of 2012, the population of VT is about 73,000 of whom 44% are migrants engaged in agriculture, mainly coca leaf cultivation [33]. The population increased by 52% between 1992 and 2012, and currently more than 400 communities are registered, 90 of which are within the park. In 2012, it was estimated that 95% of the population lived below the poverty line and 22% were in extreme poverty in terms of unsatisfactory basic living conditions [34].

The department of Cochabamba was used for the analysis of the impact on health and budget on the population-level. Cochabamba includes the municipality of VT and several other endemic rainforest areas with similar sociodemographic and geographic characteristics. We estimated the annual number of CL cases treated with SPA under current practice in Cochabamba to be 237, which corresponds to the average annual number treated over the period 2011–16 (personal communication, National Leishmaniasis Control Program (NLCP) departmental office, Novem*ber 17*^*th*^ *of 2017*). An analysis of patient records in the NLCP database for the years 2015–6 regarding the number and body location of the ulcers of all patients treated revealed that 23% had more than one lesion and 3% had lesions on the face or genitals. Data about other eligibility criteria such as ulcer size or presence of immunosuppression were not available, and therefore, we assumed that if ILPA is offered as first-line treatment, then 74% of those who seek care will receive ILPA (N = 175 if care-seeking is unchanged) and the remainder will receive SPA.

### Ethical approval and consent to participate

Approval of the study was obtained from the Ethical Committee of the School of Medicine in San Simon University. All study participants provided written informed consent after the purpose of the study was explained to them. All data was anonymised to guarantee the confidentiality and privacy of the participants. Participation in the study was voluntary, and no incentives were provided to participants.

To protect the confidentiality of patients registered in the NLCP database, personal identification information was deleted and replaced by a code.

### Study design

Two types of economic analysis were conducted: a cost analysis on a per-patient level, and a budget impact analysis on the population-level. First, a cost analysis was conducted in VT municipality and the outcome was measured in terms of cost per patient treated. All costs were assessed in Bolivian currency (BOB) and converted to international dollars (INT$) using the purchasing power parity adjusted exchange rate for 2017 (3.102 BOB = 1 INT$)[35]. Then, for the budget impact analysis, the results of the cost analysis were projected to the Cochabamba department to estimate the incremental cost of the implementation of ILPA as first-line treatment for CL at population level. The budget impact analysis was tested in four hypothetical scenarios of increase of utilisation of treatment using data from the NLCP.

Costs-per-patient treated with SPA and ILPA were estimated from the payer perspective (Ministry of Health) and from a societal perspective in which patients’ direct and indirect costs were included. Diagnostic costs were excluded since they do not differ between SPA and ILPA.

No discounting was applied to future costs or health outcomes because the follow-up period in which to consider a case cured is one year according to WHO/NTD guidelines [20]. The budget impact analysis was similarly limited to a one-year cohort of patients.

### Therapeutic alternatives to be compared

SPA is the current standard treatment for CL in Bolivia. It consists of intramuscular or intravenous antimonials administered in a dosage of 20 mg/kg per day for 20 consecutive days. Two antimonial compounds are currently available: sodium stibogluconate (SSG) and meglumine antimoniate (MA) which is used in Bolivia. SPA is frequently associated with side effects that can lead to treatment interruption and it is contraindicated in pregnant women and infants [20]. Renal, pancreatic and hepatic toxicity are life-threatening complications, more frequent in older people, which require evaluations before and during treatment via complementary exams [32]. In practice, these exams cannot be performed in most health centres in rural areas of Cochabamba due to a lack of equipment.

ILPA consists of 1 to 10 intradermal infiltrations of 1–5 mL of the same pentavalent antimonials drugs as used in SPA into the base and margins of the lesion per session for 3–7 days. The most important advantages of this treatment are the negligible side effects, the flexible schedule of administration and increased adherence by patients [20, 22].

Another alternative for treating CL, as recommended by PAHO and WHO, is thermotherapy. A previous economic evaluation of thermotherapy in Colombia found that it was cost-effective compared to SPA[63], however that analysis did not include the purchase cost of the thermotherapy equipment itself. The main reason that we do not consider thermotherapy as an alternative treatment in this paper is that purchasing new equipment is not presently a feasible option for health centres in the remote areas of Cochabamba, due to a lack of economic resources.

### Provider costs

Resource use quantities and unit prices for SPA and ILPA were estimated from several sources and are summarised in Table 1.

**Table 1:** Resource use quantities and unit prices (in INT$).

Cost item	Quantity (sensitivity analysis values)		Unit price (INT$)	Sources
	SPA	ILPA		
**Staff time use**	16 min per application (48 min)	15 min per application	1670 per month	Health facility survey and Ministry of Economy and Public Finance
**Drug applications**	20	5 (1–10)	N/A	Meta-analysis[23] Systematic review[22]
**Drug ampules**	3 per application	2 per application	4.1 per ampule (2.1–12.4)	Ministry of Health
**Surcharges for supplies**	One-off	One-off	1.0	Health facility survey
**Drugs administration fee**	20 times	5 times	0.7 per application	Health facility survey
**Transport**	20 round trips	5 round trips	3.5 per round trip (1.8–10.6)	Key informants
**Meals**	20 meals	5 meals	3.2 per meal	
**Productivity loss**	20 days (5 days)	5 days (0 days)	21.5 per day	Ministry of Economy and Public Finance

Notes to Table 1: Base case values are provided with one-way sensitivity analysis values in brackets.

The current cost of SPA was estimated using document review and a health facility survey in 12 public health facilities in VT municipality, conducted during July and August 2017. The survey included questions about the number of patients treated, costs of supplies, and the application time for administering SPA. The survey respondent was the person responsible for CL treatment in each facility.

It was assumed that ILPA would be provided at the health centre and could be administered by either nurses or health workers. The time required to apply ILPA was estimated by a key informant who is a national expert and the principal investigator of a recent study on ILPA in Bolivia (E. Rojas, personal communication, November 15^th^ of 2017).

Staff time was valued using the hourly wage of a nurse, assuming 160 working-hours per month. Salary scales for physicians, nurses, and health workers for the year 2017 were obtained from the Ministry of Economy and Public Finance [37].

The dosage for SPA was based on the NLCP’s at 20mg / kg / day for 20 days [3, 21]. There are no standard guidelines regarding the number of ILPA infiltrations to be applied. We used the average number of infiltrations (n = 5) and the maximum number of ampules needed given the maximum doses applied in all the studies included in a recent meta-analysis [22] (2 ampules equivalent to 10 ml per infiltration).

The cost per 5 ml ampule was obtained from the departmental office of the NLCP in Cochabamba city [38] and corresponds to the last drugs purchase in November 2017.

We included the cost of supplies in the societal, but not in the MoH perspective, because in this setting, supplies are paid out-of-pocket by patients.

### Patient costs

It was not feasible to conduct a patient survey because CL cases are very dispersed among the communities in the study area and sporadic across time. Instead, patient out-of-pocket expenses were estimated using the health facility survey and from key informants. Surcharges for supplies such as syringes and disinfectant, which are paid for by patients, and drugs administration fees, were obtained from the health facilities survey and multiplied by the number of treatment applications. The cost of transport was estimated through key informant interviews with members of the public transport labour union of the VT municipality during July and August 2017. The informants were asked about travel times and costs to the health centres from the communities which reported the highest number of CL cases to NLCP in the last two years. The daily cost of meals was estimated through personal communication with field workers affiliated with the Tropical Medicine Centre at San Simon University.

The time cost for households was estimated assuming that the number of working days lost due to illness was equal to the treatment duration. This productivity loss was valued using the Bolivian minimum monthly wage for 2017 from the Ministry of Economy and Public Finance, 644.7 INT$, assuming 160 working hours per month.

### Sensitivity analysis

One-way sensitivity analysis was performed to evaluate the effect of parameter uncertainty on the cost per patient treated, from the MoH and societal perspectives. The parameters evaluated were staff time use (SPA only), the number of ILPA infiltrations, the price of drugs, the number of ampules (ILPA only), transport cost, and number of lost working days (Table 1). For the staff time, we considered a time increase of 200% for SPA. This time is needed for some patients who develop adverse reactions without indications of treatment interruption. The minimum and maximum number of infiltrations described in the studies included in the meta-analysis was used as reference for the number of infiltrations [22]. A variation of 50%-200% was considered to reflect the changes observed in recent years for drugs price. Finally, an incremental cost of 50%-200% for the cost of transport, which usually happens when strong rains damage the roads, was considered. A productivity loss ranging from 5 to 30 days was considered for SPA because, the duration of productivity loss varies depending on the severity of adverse events and the ulcer’s time to heal. A reduction in productivity loss to 0 days was considered for ILPA because some ulcers are very small and ILPA does not affect a patient’s physical activity.

### Budget impact analysis

Four scenarios were constructed with which to evaluate the budget impact compared to current practice:

- An increase in treatment utilisation of 20% of reported cases using only SPA.
- Current treatment utilisation using ILPA as first line treatment;
- An increase in treatment utilisation of 20% of reported cases using ILPA as first line treatment;
- An increase in treatment utilisation to 80% of total expected cases using ILPA as first line treatment;

We assumed that all cases were eligible for SPA since in practice, providers do not undertake analyses to identify potential toxicity to SPA.

Scenario 1 corresponds to an increase of 20% to the average treatment utilisation only using SPA. Scenarios 2, 3 and 4 were constructed based on the assumption of a change using ILPA as the first-line treatment. Scenario 2 corresponds to the average treatment utilisation in Cochabamba reported by NLCP from 2011 to 2016; Scenario 3 corresponds to the same increase in treatment utilisation as scenario 1; Scenario 4 corresponds to an assumed increase in treatment utilisation of up to 80% of the total cases expected, not only those reported. This distinction is important because very low levels of access to treatment (26.6%) were found in a previous study in the region [39]. Using the results of that study, and the reported cases by NLCP (n = 237), we estimated that there could be a total of up to 891 cases (237/0.266) in Cochabamba per year.

## Results

ILPA was cost saving compared to SPA, from both the MoH and the societal perspective. For the MoH, the cost of treating a patient with SPA was 303.5 INT$ for SPA and 55.2 INT$, for ILPA, a net difference in favour of ILPA of 248.2 INT$. The main contribution to cost saving was the large reduction in the quantity of drugs used (83%). From the societal perspective, the cost of SPA was 889.5 INT$ per patient for SPA and 201.7 INT$ for ILPA, a net difference in favour of ILPA of 687.8 INT$. The drug cost reduction represented 30% of the total societal cost savings, and nearly half (47%) was related to fewer working days lost during the duration of treatment (Table 2).

**Table 2:** Costs per patient treated (2017 INT$).

Cost components	SPA	ILPA	Incremental cost of ILPA
Staff time cost	55.7	13.9	-41.7
Drug cost	247.8	41.3	-206.5
**Sub-total MoH costs**	**303.5**	**55.2**	**-248.2**
Supplies (out-of-pocket payment)	20.6	5.2	-15.5
Meals	64.5	16.1	-48.4
Transport	70.9	17.7	-53.2
Productivity loss	430.0	107.5	-322.5
**Sum societal costs**	**889.5**	**201.7**	**-687.8**

For SPA, the total out-of-pocket payments reached 170.9 INT$, of which 21% was due to surcharges related to supplies and drug administration fees. The total savings in out-of-pocket payments for patients treated with ILPA was 128.1 INT$ compared to those treated with SPA (Table 3).

**Table 3:** Household cost components per patient treated (2017 INT$).

Household costs components	SPA	ILPA	Incremental cost of ILPA
Drugs administration fee	14.8	3.7	-11.1
Surcharges for Supplies	20.6	5.2	-15.5
Meals	64.5	16.1	-48.4
Transport	70.9	17.7	-53.2
**Sum household costs**	**170.9**	**42.7**	**-128.1**

### One-way sensitivity analysis

**Fig 1:**
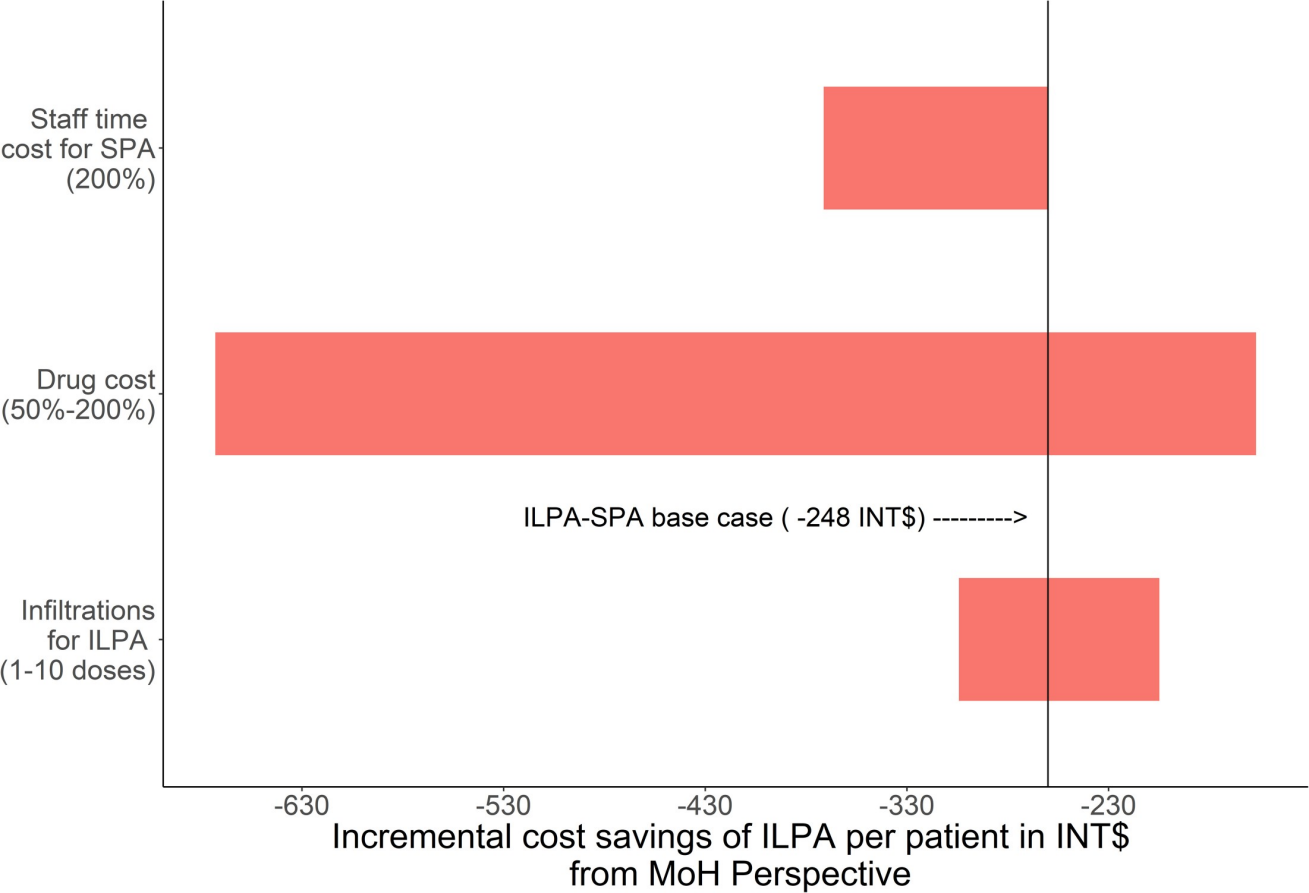
Tornado diagram of one-way sensitivity analyses of basic assumption effects on the incremental cost of ILPA expressed in INT$ from the MoH perspective.

In the one-way sensitivity analysis from the MoH perspective (Fig 1), ILPA remained cost saving compared to SPA across all variations of parameters including the number of ILPA infiltrations, the cost of the drugs, and staff time to administer SPA.

**Fig 2:**
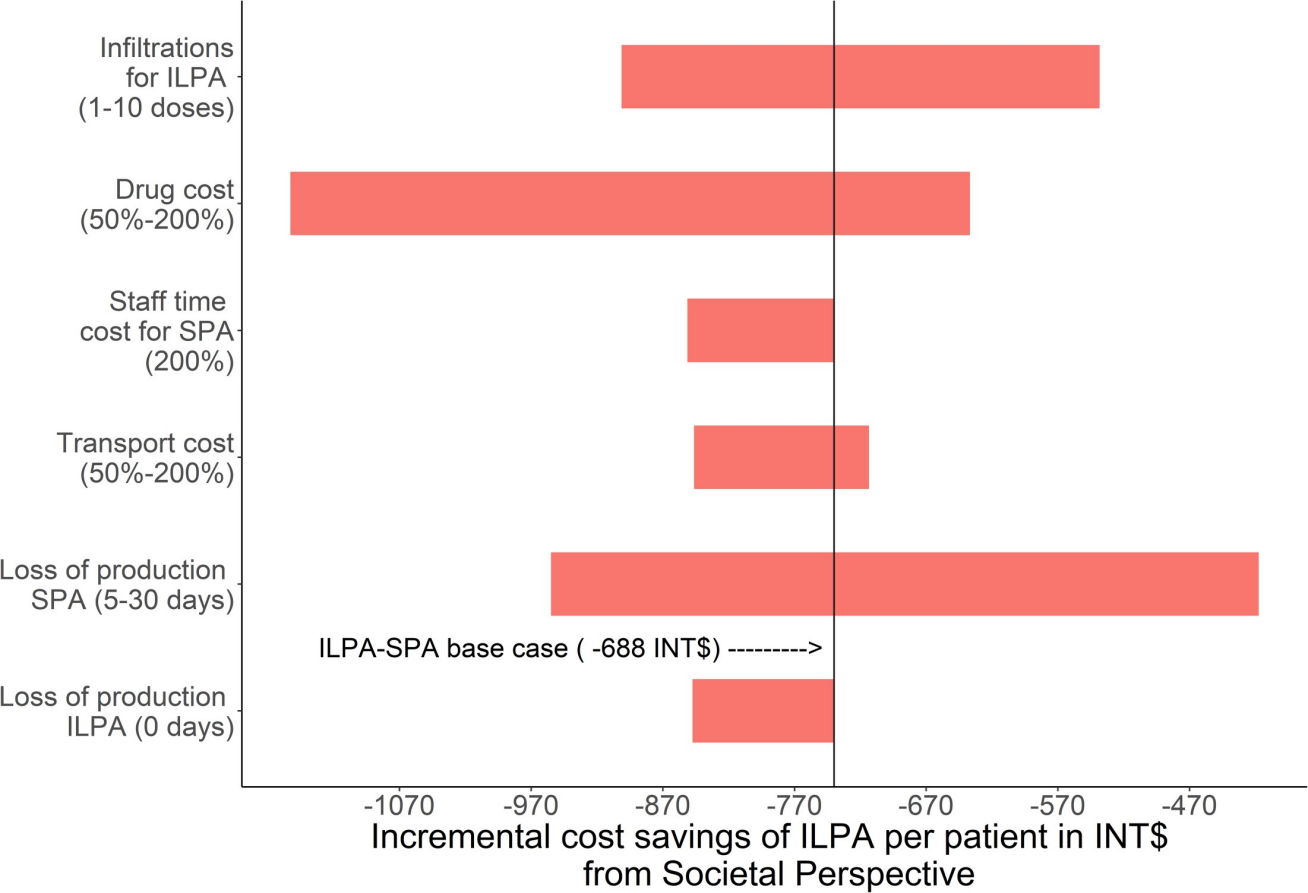
Tornado diagram of one-way sensitivity analyses of basic assumption effects on the incremental cost of ILPA expressed in INT$ from a societal perspective.

Similar findings were found from the societal perspective. The size of the cost saving was more strongly affected by drug costs, the number of infiltrations with ILPA, and the number of working days lost when receiving SPA. The result was less sensitive to staff time, transport cost, and working days lost when receiving ILPA (Fig 2).

### Budget impact and health improvement using ILPA as the first line treatment for CL

The cost of SPA for the 237 cases reported annually reached 71,906 INT$ for the MoH. Considering a modest 20% increase in treatment utilisation keeping SPA as the only treatment results in an increased expenditure of 14,381 INT$ (Scenario 1). Changing to ILPA as first line of treatment, assuming that treatment utilisation remains at the current level, would represent a saving of 43,529 INT$ (Scenario 2), while the same increase in treatment utilisation as Scenario 1 but using ILPA as the first-line treatment represents a cost saving of 37,854 INT$ (Scenario 3). If treatment utilisation increases to 80% of expected cases (three times the current number of cases treated), an increase in the budget of 13,437 INT$ would be required (Scenario 4).

Retaining the current budget but using ILPA as the first-line treatment could allow the treatment of 601 patients instead of the current 237 patients (Table 4).

**Table 4:** Budget impact analysis from the MoH perspective (INT$).

Scenarios	Number of cases treated			MoH perspective	
	SPA	ILPA	Total	Cost	Budget Impact
**Current Practice**	237	0	237	71,906	0.0
**1) 20% increase in utilisation on reported cases†**	284	0	284	86,287	14,381
2) Current level of treatment utilization*	62	175	237	28,376	-43,529
3) 20% increase in utilisation on reported cases*	74	210	284	34,052	-37,854
4) Increase in treatment utilisation to 80% of total expected cases*	185	527	713	85,343	13,437
Total patients can be treated with the current budget*	156	444	601	71,906	0.0

Notes to Table 4

† Current practice, SPA only.

* Using ILPA as first-line treatment

## Discussion

This study illustrates the reality of treatment in the country with the fifth highest number of CL cases in the Americas and demonstrates the economic and health benefits of switching from SPA to ILPA as the first-line treatment. Through reducing the costs per patient treated, switching to ILPA would allow more patients to be treated within the current budget, as well as reduce the adverse effects associated with SPA.

In terms of efficacy, evidence from systematic reviews and meta-analysis shows no difference between the two treatments (75% for SPA [23] and 77% for ILPA)[22]. However, some international guidelines suggest avoiding the use of ILPA among CL cases caused by *Leishmania* species when there is a risk of mucosal involvement, such as in the case of the *Leishmania* species of the *Viannia* subgenus *(L*. *braziliensis*. *L*. *guyanensis*. *L*. *amazonensis and L*. *panamensis)* [40, 41]. On the other hand, there are reports of good efficacy in Brazil [25, 42–45] and Bolivia [46, 47] where the majority of CL cases are produced by *L*. *brazilensis*. Regarding mucosal complications, two studies in Brazil that conducted a five-year follow-up of patients treated with ILPA did not find evidence of relapses or mucosal leishmaniasis [28, 29].

Leishmaniasis is a complex disease in which the clinical response to the treatment is related to several factors linked to the host, the drug, and the parasite. There is evidence that SPA does not completely clear parasites from the body [48, 49]. In fact, the presence of *Leishmania* in mucosal tissues in the apparent absence of disease seems to be a common feature of the natural history of infection due to *Leishmania* species of the *Viannia* subgenus [50–53]. The metastasis to mucosal tissues per se does not explain the pathogenesis of mucosal leishmaniasis, which supports the idea that host response plays a role in the pathogenesis of mucosal disease. In fact, it is well-known that the immunomodulatory effects of antimonials, enhancing monocyte functions by directly increasing phagocytosis [54–56], play a key role against the parasite in human leishmaniasis, equally important than its leishmanicidal activity. That is why when immune function is diminished, the treatment response is poor, there is a high rate of recurrence and risk of dissemination, and subsequently, higher probability of mucosal complications [54, 57]. ILPA also produces immunomodulatory activities [58, 59] and systemic distribution in the body [60], which could explain the absence of mucosal complications reported with this treatment.

Economic studies are important in order to evaluate the transferability of results from clinical trials to real-world scenarios. Only a handful of economic studies have evaluated the cost-effectiveness of SPA and other new therapeutic alternatives for CL, and none have focused on ILPA. One study from Afghanistan assessed the cost effectiveness of SPA in terms of Disability Adjusted Life Years (DALY) averted, and concluded that using SPA to treat CL was not cost effective, using thresholds based on Gross Domestic Product from WHO-CHOICE [61]. A study conducted in Colombia found that in paediatric patients, miltefosine was slightly more expensive than SPA from a government payer perspective but substantially less expensive from the societal perspective due to savings related to the costs of food, lodging, transportation and productivity loss [62]. Similar results were found in our study where the highest savings using ILPA were related to reductions in the costs of meals, transportation and productivity loss. A third study, also conducted in Colombia from the government perspective, found that thermotherapy, the only other alternative to ILPA currently recommended by PAHO to treat CL [63], was cost-effective and less toxic for treating CL compared to SPA. The cost saving of thermotherapy was related mainly to a reduction in complementary examinations and the treatment of associated adverse reactions (USD 167 per patient). One major limitation in this study, however, was the omission of the purchase cost of the thermotherapy equipment.

### Methodological considerations

Since ILPA is not currently provided in the area we studied, and no standardized scheme exists for its administration, several assumptions about how ILPA would be administered had to be made. Specifically, the volume and dosage were based on mean values reported in systematic reviews and might not correspond exactly to a standardized scheme, were such to be introduced, nor to observed values, if the treatment was to be offered as part of routine practice in this context.

Although the current standard SPA treatment can have serious adverse effects, our study did not include costs related to treatment of adverse reactions, nor the costs of control laboratory analysis and electrocardiograms during treatment. This decision reflects the practical situations of health centres in remote rural areas of Bolivia in which there are no means to treat the toxicity of these drugs, nor the equipment to perform these examinations. Their inclusion in the analysis would increase the cost-savings associated with ILPA.

Household cost were estimated for the specific Bolivian context and can vary in other settings. Household costs were limited to direct healthcare payments, meals, and transport, which are likely the major household cost items in this context. Including other household costs such as for childcare or sub-contracting of workers, would further increase the estimated cost difference between SPA and ILPA.

To increase the relevance and value of primary research studies for decision-makers, it is important that future studies of ILPA and other CL treatment alternatives collect cost data from the household as well as from the provider perspectives.

The specific susceptibility of the different species of *Leishmania* was not considered; however, it is well known that *L*. *braziliensis* causes more than 85% of the cases in Bolivia [64] and has a very good response to antimonials [65, 66].

### Conclusions and policy implications

This study demonstrates that ILPA is most likely cost-saving and affordable compared to SPA in the context of the Bolivian rainforest. Changing to ILPA as the first-line treatment is therefore an opportunity for the MoH to treat more patients within the current budget. This policy change would also directly address the resource constraints of primary health centres in remote areas, where SPA cannot be provided in safe conditions.

The adoption of ILPA in Bolivia as a first line treatment for CL could have a direct impact in reducing direct and indirect financial barriers to accessing CL treatment, and thus contribute to better control of the disease. The implementation of ILPA would contribute to solving three main critical barriers that stop CL patients seeking and receiving treatment. First, the use of ILPA could reduce periods of drug shortages related to the budget constraints of the health system. Secondly, from a patient’s perspective, it would mean less loss of work and smaller out-of-pocket costs. Thirdly, due to fewer adverse reactions and the absence of toxicity, the use of ILPA would likely result in better case management and disease control, in particular if the shorter treatment duration improves adherence to treatment.

It is our hope that this study will encourage decision-makers to urgently include ILPA as a first-line treatment in the national leishmaniasis treatment guidelines. This policy change will give a therapeutic alternative to remote rural areas of the Latin American rainforest where CL is an endemic problem and health services cannot provide SPA in safe conditions. Furthermore, the implementation of ILPA jointly with the SPA must be considered as a possible solution to face the difficulties of access to treatment and availability of the specific drug for CL cases.

## Supporting information

S1 Data Microsoft Excel document comprising survey answers, data from official documents, as well as the cost and budget impact analysis. (XLSX)

S1 Table CHEERS checklist. (DOCX)
